# Impact of follow-up liver biopsy on long-term outcomes post-Kasai procedure in patients with biliary atresia

**DOI:** 10.1007/s00383-025-05979-y

**Published:** 2025-02-28

**Authors:** Koki Takase, Takehisa Ueno, Sayaka Matsumoto, Naoko Uga, Koichi Deguchi, Motonari Nomura, Miho Watanabe, Masafumi Kamiyama, Yuko Tazuke, Takeshi Kimura, Hiroomi Okuyama

**Affiliations:** 1 Department of Pediatric Surgery, Osaka University of Graduation School of Medicine, 2-2 Yamadaoka, Suita, Osaka 565-0871 Japan; 2 Department of Pediatrics, Osaka University of Graduation School of Medicine, 2-2 Yamadaoka, Suita, Osaka 565-0871 Japan

**Keywords:** Biliary atresia, Liver biopsy, Long-term survival, Liver transplantation

## Abstract

**Purpose:**

Patients with biliary atresia (BA) suffer from progressive liver damage, even after successful Kasai portoenterostomy (KPE). The purpose of this study is to analyze the relevance of follow-up percutaneous liver biopsy (LBx) and long-term prognosis of patients with BA.

**Methods:**

This study included patients with BA who were born between 1983 and 2005 and survived with their native liver until 10 years of age. Patient characteristics, laboratory data and Child–Pugh score at the time of LBx, and native-liver survival (NLS) and complication-free survival (CFS) in patients with mild (F0-F2) or severe fibrosis (F3, F4) on follow-up LBx were retrospectively analyzed.

**Results:**

Forty-three patients were gathered in this study and the most recent LBx was performed at age 21.1 ± 2.9 years. Thirty-three patients had mild fibrosis and ten patients had severe fibrosis on follow-up LBx. Long-term NLS and CFS were significantly worse in patients with severe fibrosis. Among those patients, 18 patients had follow-up LBx between the ages of 6 and 12 years, and CFS were significantly worse in patients with severe fibrosis.

**Conclusions:**

We found that patients with BA with severe liver fibrosis on follow-up LBx had worse long-term survival and a higher rate of progression of complications of BA.

## Introduction

Patients with biliary atresia (BA) suffer from progressive liver damage, even after successful Kasai portoenterostomy (KPE) [1–3]. One-third of patients with BA experience rapid deterioration of liver function and require liver transplant (LT) before 2 years of age [3]. Among the remaining patients with BA, many patients have gradual, indolent progression of liver fibrosis and experience complications such as cholangitis and portal hypertension [4, 5]. The Japanese Biliary Atresia Registry reported that 49% of patients with BA require LT before the age of 20 years [3].

Liver biopsy (LBx) is performed to evaluate the severity of liver fibrosis in patients with BA [6]. Although the relationship between LBx at the time of KPE and patient outcomes has been reported, there is no literature about follow-up LBx after KPE and patient long-term outcomes.

Here, we report the relationship between liver fibrosis on follow-up LBx and long-term outcomes in patients with BA post-KPE.

## Methods

### Patients

This study included adult patients older than 18 years old with BA who were born between 1983 and 2005 and survived with their native liver until 10 years of age. Follow-up percutaneous LBx was performed to evaluate the current status of liver fibrosis or cirrhosis as previously reported, basically every year by 6-year-old, every 2 years by 12-year-old, and every 3 to 5 years thereafter, depending on their degree of liver fibrosis and complications of BA [7]. Once follow-up LBx demonstrated cirrhosis, further follow-up LBxs were not performed. Medical records and laboratory findings were retrospectively reviewed. The patients’ characteristics (age, sex, age at liver biopsy) and their outcomes and complications were retrieved. Complication-free survival (CFS) was defined as survival without complications including an indication for liver transplant (e.g., liver failure, refractory cholangitis, refractory gastrointestinal bleeding due to esophagogastric or intestinal varices, hepatopulmonary syndrome, or porto-pulmonary hypertension) [8]. Splenomegaly and gastroesophageal varices were defined those that was detected by abdominal ultrasound or computed tomography, and by esophagogastroduodenal endoscopy, respectively. The patients’ age, survival, and complications were assessed in March 2023.

### Follow-up biopsy

Specimens gathered from the follow-up biopsy of patients with their native liver were assessed for pathologic liver fibrosis. For patients who had received liver transplantation, the LBx data before the time of transplantation was used. LBx samples were assessed with hematoxylin–eosin and Masson’s trichrome stains. All biopsies were performed under either general anesthesia or local anesthesia with sedation. LBxs were taken percutaneously with a 16-gage biopsy needle from an avascular area under ultrasound. Specimens were fixed in 4% phosphate-buffered formaldehyde and embedded in paraffin. After hematoxylin–eosin and Masson’s trichrome staining, LBx specimens were examined microscopically by experienced pathologists in our hospital. The liver fibrosis score was graded by METAVIOR score (F0–F4), and we defined F0, F1, and F2 as mild fibrosis and F3 and F4 as severe fibrosis [9].

### Data analysis

The laboratory data were compared with the LBx results. The data included alanine aminotransferase (ALT), gamma-glutamyltranspeptidase (GGTP), total bilirubin (T-Bil), total bile acid (TBA), serum albumin (Alb), choline esterase (ChE), prothrombin time (international normalized ratio, PT-INR), platelet count (Plt), Child-Pugh score and Pediatric End-stage Liver Disease (PELD) score at the time of liver biopsy. PELD score was calculated using the following equation: PELD = 4.80[Ln{serum bilirubin (mg/dL)}] + 18 .57[Ln{INR}] − 6.87[Ln{albumin (g/dL)}] + 4.36( < 1 year old) + 6.67(growth failure) [7].

During prognosis analysis, the prognostic predictability of liver fibrosis was assessed. Patients who had follow-up LBx at aged 6–12 years were included and patients whose Child-Pugh score exceeded class A at the time of LBx were excluded. The relationship between pathologic liver fibrosis and long-term outcomes was analyzed as described earlier.

### Statistical analysis

Continuous variables were expressed as mean ± 2SD and analyzed using the two-sample t-test. Categorical variables were analyzed using Pearson’s Chi-square test. A difference was considered significant when *p* < 0.05. Survival assessment was carried out using the Kaplan–Meier curve method and log-rank analysis. For the analysis of native-liver survival (NLS), survival started at birth and was censored at the time of LT or death. For the analysis of CFS, survival also started at birth and was censored at the time of detection of complications or consideration of LT. Statistical analyses were performed with JMP Pro 16 software (SAS Institute, Cary, NC, USA).

This study was approved by our hospital’s institutional review board (approval number 23359). The study was performed according to the ethical standards of the 1964 Declaration of Helsinki and its later amendments. All patients or their guardians gave informed consent prior to their inclusion in this study.

## Results

### Characteristics of the study patients

Demographic characteristics of the study patients are summarized in Table 1. Forty-three patients were included in this study. The mean age of the patients was 30.2±2.0 years and the most recent LBx was performed at age 21.1±2.9 year. Among these 43 patients, 26 patients had survived with their native liver, including 4 patients with complications: liver failure (n=2), gastrointestinal bleeding (n=1), and pulmonary hypertension (n=1). LT was performed in 13 patients, and for these patients, LBx results before the time of transplantation were used for assessment. Four patients had died without receiving LT. CFS was achieved in 22 patients. The number of patients at each liver fibrosis stage on the latest biopsy were F0 = 7, F1 = 18, F2 = 8, F3 = 5, and F4 = 5. 32 (74%) out of the 43 cases had serial liver biopsies performed more than once. In the severe group, whose last available was F3-4, 7 out of 10 patients had a previous biopsy, and 4 (57%) of 7 cases progressed from the mild group (F0-2) in the previous biopsy to the severe group (F3-4) in the last biopsy.

**Table 1:** Patient demographics and laboratory data, Child–Pugh score of all patients

	All patients	Mild fibrosis	Severe fibrosis	*p* value*
Age (years)*	30.2 ± 2.0	32.4 ± 1.6	24.5 ± 3.6	0.009
Sex M/F	14/29	10/23	4/6	N.S
NLS/ liver transplantation/ death without liver transplantation	26/13/4	23/7/3	3/6/1	N.S
Complications: Liver failure/GI bleeding/pulmonary hypertension	2/1/1	1/0/1	1/1/0	N.S
Splenomegaly*	70.0%	61.3%	100%	0.037
Gastroesophageal varices	45.9%	37.9%	75%	N.S
LBx data				
Age at liver biopsy (years)*	21.1 ± 2.9	23.0 ± 3.0	14.4 ± 3.1	< 0.001
Liver fibrosis: F0/F1/F2/F3/F4	7/18/8/5/5			
Laboratory data at the times of LBx				
ALT (U/l)	56.1 ± 13.7	45.5 ± 11.0	91.3 ± 35.2	N.S
GGTP (U/l)	147.3 ± 46.3	126.4 ± 47.1	214.2 ± 101.3	N.S
T-Bil (mg/dl)	2.0 ± 0.6	1.6 ± 0.5	3.3 ± 1.4	N.S
TBA (mg/dl)	64.1 ± 27.1	51.2 ± 28.7	126.4 ± 53.7	0.040
Alb (g/dl)	3.8 ± 0.2	3.9 ± 0.2	3.3 ± 0.4	0.007
ChE (U/l)	212.3 ± 26.9	229.3 ± 31.4	157.8 ± 35.1	0.006
PT-INR	1.15 ± 0.04	1.12 ± 0.04	1.24 ± 0.05	0.008
Plt (× 10^4^/μl)	13.0 ± 2.5	14.8 ± 2.9	6.9 ± 2.9	< 0.001
Liver failure at the times of LBx				
Child–Pugh score	5.9 ± 0.4	5.7 ± 0.4	6.5 ± 0.7	N.S

^*^p value between patients with mild and severe fibrosis

*SL* serum albumin, *ALT* alanine aminotransferase, *CFS* complication-free survival, *ChE* cholinesterase, *F* female, *GGTP* gamma-glutamyltranspeptidase, *LBx* liver biopsy, *M* male, *NLS* native-liver survival, *N.S.* not significant, *Plt* platelet count, *PT-INR* prothrombin time international normalized ratio, *TBA* T-Bil total bilirubin

The patient demographics and laboratory data by pathologic findings are also shown in Table 1. The patient age and age at the latest LBx were lower in patients with severe fibrosis. For safety reasons, an additional serial biopsy was not performed once the patient reached F4 fibrosis in this study. Patients with severe fibrosis may also have had a liver transplant before age 10. Therefore, the age of last biopsy appeared to be younger in severe fibrosis than that of mild fibrosis. The incidence of complications of portal hypertension, such as splenomegaly and gastroesophageal varices were high in patients with severe fibrosis, and the difference was significant for splenomegaly. TBA, and PT-INR were significantly higher and Alb, ChE, and platelets were significantly lower in patients with severe fibrosis. There was no difference in Child-Pugh scores between patients with mild and severe fibrosis.

### Characteristics of the patients with school-age biopsy

Eighteen patients had follow-up LBx between the ages of 6 and 12 years and Child-Pugh class A (C-P A) at the time of liver biopsy. Demographic characteristics and laboratory data at the time of LBx for the study patients are summarized in Table 2. The mean age of the patients was 26.9±3.1 years and the LBx was performed at the age of 10.4±0.6 years. Thirteen patients had survived with their native liver, including three patients with complications (liver failure one, gastrointestinal bleeding one, and pulmonary hypertension one). LT was performed in four patients and one patient died without receiving LT. CFS was achieved in ten patients. The number of patients with each liver fibrosis stage on the biopsy was as follows: F0 = 4, F1 = 4, F3 = 5, F3 = 2, and F4 = 2.

**Table 2:** Patient demographics and laboratory data, Child–Pugh score of in patients with follow-up liver biopsy between the ages of 6 and 12 years

	School-age, Child–Pugh A	Mild fibrosis	Severe fibrosis	*p* value*
Age (years)*	26.9 ± 3.1	28.6 ± 3.3	20.9 ± 2.7	0.003
Sex M/F	7/11	6/8	1/3	N.S
NLS/ liver transplantation/ death without liver transplantation	13/4/1	11/2/1	2/2/0	N.S
Complications: Liver failure/GI bleeding/pulmonary hypertension	1/1/1	0/1/1	1/0/0	N.S
LBx data				
Age at liver biopsy (years)	10.4 ± 0.6	10.3 ± 0.7	11.0 ± 0.5	N.S
Liver fibrosis: F0/F1/F2/F3/F4	4/5/5/2/2			
Laboratory data at the times of LBx				
ALT (U/l)	62.8 ± 23.5	60.7 ± 28.5	70.3 ± 41.2	N.S
GGTP (U/l)	155.9 ± 91.7	139.6 ± 111.7	209.0 ± 153.8	N.S
T-Bil (mg/dl)	1.1 ± 0.1	0.9 ± 0.2	1.7 ± 0.7	N.S
TBA (mg/dl)	52.2 ± 31.6	28.2 ± 20.0	106.2 ± 70.4	N.S
Alb (g/dl)	4.0 ± 0.2	4.1 ± 0.2	3.6 ± 0.3	0.022
ChE (U/l)	252.6 ± 35.0	267.8 ± 41.0	203.5 ± 40.9	N.S
PT-INR	1.15 ± 0.07	1.16 ± 0.09	1.11 ± 0.07	N.S
Plt (× 10^4^/μl)	13.0 ± 3.7	14.7 ± 4.4	7.6 ± 3.5	0.025
Liver failure at the times of LBx				
Child–Pugh score	5.3 ± 0.2	5.2 ± 0.2	5.5 ± 0.6	N.S

^*^p value between patients with mild and severe fibrosis

*Alb* serum albumin, *ALT* alanine aminotransferase, *CFS* complication-free survival, *ChE* cholinesterase, *F* female, *GGTP* gamma-glutamyltranspeptidase, *LBx* liver biopsy, *M* male, *NLS* native-liver survival, *N.S.* not significant, *Plt* platelet count, *PT-INR* prothrombin time international normalized ratio, *TBA* total bile acid, *T-Bil* total bilirubin

Patient demographics and laboratory data by pathologic findings are also shown in Table 2. The patients with severe fibrosis were younger, and Alb and platelets were significantly lower in patients with severe fibrosis. There was no difference in Child-Pugh scores and PELD scores between patients with mild and severe fibrosis.

### Kaplan–Meier curve and log-rank analysis for NLS and CFS by pathologic findings

**Fig. 1:**
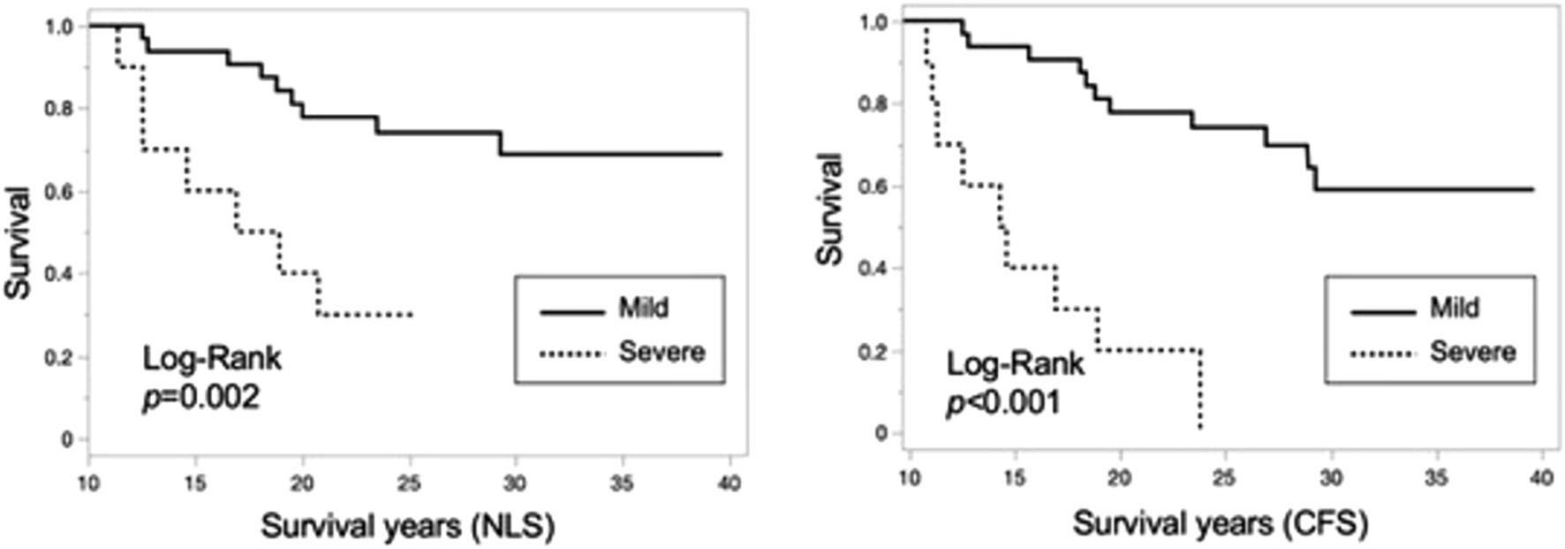
Kaplan–Meier curve and log-rank analysis for NLS and CSF in patients with mild or severe fibrosis. *CFS* complication-free survival, *NLS* native-liver survival, *N.S.* not significant

**Fig. 2:**
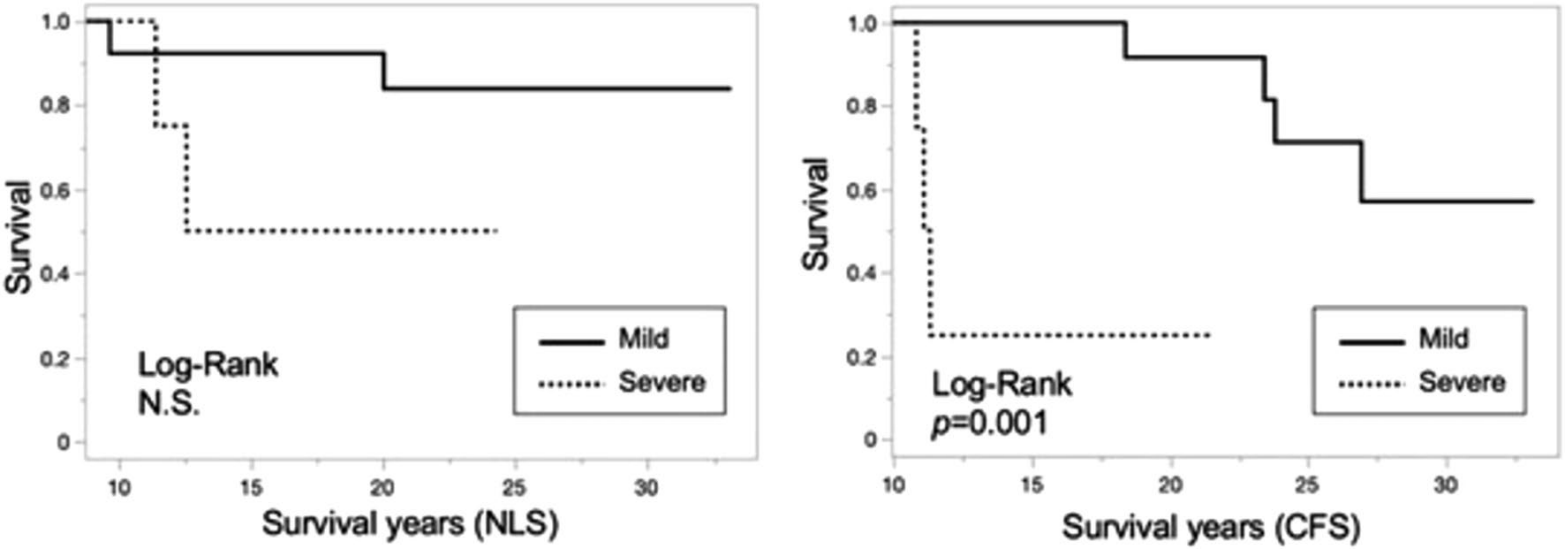
Kaplan–Meier curve and log-rank analysis for NLS and CSF in patients with mild or severe fibrosis among patients with school-age biopsy. *CFS* complication-free survival, *NLS* native-liver survival, *N.S.* not significant

The Kaplan–Meier curve and the results of the log-rank analysis of patient survival are shown in Figs. 1 and 2. Among all patients, NLS and CFS were significantly worse in patients with severe fibrosis (Fig. 1). Despite NLS in patients who had follow-up LBx between the ages of 6 and 12 years did not differ between patients with mild and severe fibrosis, CFS were significantly worse in patients with severe fibrosis (Fig. 2).

## Discussion

In patients with BA who survive with their native liver until adolescence or adulthood, it is difficult to assess the necessity and appropriate timing of LT, due to the slow progression of complications [10]. While some patients have a very good prognosis and do not experience jaundice or any complications or progression of liver fibrosis, other patients develop progressive liver fibrosis and cirrhosis, experience complications, and require the consideration of LT, even though they could survive with their native liver until adulthood [4, 11]. In a previous report of living-donor liver transplant (LDLT) in Japan, the long-term graft survival was worse in recipients aged older than 12 years, and was worst in recipients aged 12–18 years [12]. In addition, there is a shortage of brain-dead donors in Japan [13]; therefore, many adult patients with BA are unable to undergo LT at the time they require it, because of the aging of their related donor candidate, or graft-size mismatch [14]. These situations make it difficult to decide when or whether LT should be performed in patients with BA who survive with their native liver until adolescence.

Our data show that patients with severe liver fibrosis or cirrhosis on follow-up LBx had significantly worse NLS and CFS. Although NLS was not differ between mild and severe fibrosis in patients who had follow-up LBx taken between the ages of 6 and 12 years, CFS in these patients was significantly worse in patients with severe fibrosis, which suggests earlier development of complications of BA. There are two reason why NLS in patients who had LBx between the ages of 6 and 12 years had not significant difference between patients with mild and severe fibrosis. First, the number of these patients was small in the current study. Second, some of these patients who had developed complications of BA were considered for LDLT but could not undergo the procedure because of the lack of suitable related donor candidates. Our data suggests that most patients with BA who have severe liver fibrosis or cirrhosis would require LT or experience complications related to BA, even if they had little or no complications at the time of LBx.

We emphasize that most patients who were graded as F3 or F4 at LBx would suffer from complications of BA during the follow-up period later. In our cohort of school-age LBx, whole patients were graded in Child-Pugh class A and had very low PELD scores, which were generally not considered to be indicated for LT [15]. Especially in Japan, there are few cadaveric donations. Given the current situation of donor shortage and requirement of LDLT for those patients, patients with BA who had severe fibrosis or cirrhosis on LBx should be considered and evaluated for LDLT in the near future, according to the condition of each patient.

The relationship between liver fibrosis and long-term prognosis has been previously reported. However, the liver fibrosis assessed was limited at the time of diagnosis of BA or KPE [6, 16–18]. Czubkowski et al. reported lower rates of NLS 10 years post-KPE in patients with severe pathologic fibrosis, but the difference was not significant [16]. Higashio et al. reported a significantly lower NLS of 15 years in patients with severe pathologic fibrosis [17]. The current study is the first report about the relationship between the post-Kasai percutaneous follow-up liver biopsy and the long-term prognosis of patients with BA.

Percutaneous liver biopsy is invasive and major complications such as severe pain, hemorrhage, organ injury or even death has been reported [19–21]. There could be higher risks of bleeding in patients with severe liver fibrosis or cirrhosis [21]; therefore, we confirm the diagnosis of cirrhosis in patients who have obtained the result of F4 in 2 separate LBxs, and do not perform LBx after the diagnosis. Since most patients with severe liver fibrosis suffered from complications of BA during the follow-up period, even though their Child-Pugh score and PELD score were low at the time of LBx, we considered that the benefits of detecting liver fibrosis with LBx outweigh the risks of LBx.

Our study has some limitations. First, the study was a retrospective, single-institute study and the number of participant patients was small. Patients with severe fibrosis were younger than those with mild fibrosis because our institute’s LT program started in 1998; therefore, more severe patients were referred to our institute following the program’s initiation. Second, the specimen of the percutaneous LBx did not correctly represent the true fibrotic stage of the whole liver. We treat serum Mac-2 binding protein index (M2BPGi) as an important marker of liver fibrosis in patients with BA [7]; however, we could not include M2BPGi, a relatively new laboratory finding, in the current study. Third, the timing of liver biopsy varies depending on the patient. Patients from school age to adolescents are often busy with school and work. The timing of liver biopsy is subject to change, as we consider the timing of liver biopsy according to the patient’s social situation on the premise that pathologic liver fibrosis does not progress rapidly. Fourth, sampling error may occur in core needle libre biopsy. In our cases, F0 at the latest liver biopsy, two patients suffered from liver failure later. The reason may be that the liver biopsies may only be taken from regenerated nodules and may result in F0 fibrosis.

In conclusion, we found that patients with BA with severe liver fibrosis on follow-up LBx had worse long-term survival and a higher rate of progression of complications of BA, even though they had little or no symptoms at the time of LBx. These results suggest that patients with severe liver fibrosis, namely F3 or F4, should be considered or prepared for LT according to their clinical situation and social situation of donor availability. Follow-up LBxs were useful for determining the patients’ prognosis.

## Data Availability

No datasets were generated or analysed during the current study.
